# 巴利昔单抗短期替代钙调蛋白抑制剂预防急性移植物抗宿主病的探索性研究

**DOI:** 10.3760/cma.j.cn121090-20230519-00201

**Published:** 2024-02

**Authors:** 珊 邵, 慧霞 刘, 瑛 蒋, 肃 李, 道林 魏, 骏 朱, 椿 王, 初娴 赵

**Affiliations:** 上海闸新中西医结合医院骨髓移植科，上海 200040 Department of Hematopoietic Stem Cell Transplantation, Shanghai Zhaxin Traditional Chinese and Western Medicine Hospital, Shanghai 200040, China

**Keywords:** 重组抗CD25人源化单克隆抗体, 异基因造血干细胞移植, 钙调蛋白抑制剂, 移植物抗宿主病, Recombinant humanized anti-CD25 monoclonal antibody, Allogeneic hematopoietic stem cell transplantation, Calcineurin inhibitors, Graft versus host disease

## Abstract

**目的:**

探讨在钙调蛋白抑制剂（CNI）不耐受患者中短期应用重组抗CD25人源化单克隆抗体巴利昔单抗（Basiliximab）单药预防异基因造血干细胞移植（allo-HSCT）后急性移植物抗宿主病（GVHD）的有效性。

**方法:**

本研究纳入2021年8月至2022年8月在上海闸新中西医结合医院骨髓移植科行挽救性allo-HSCT后因CNI严重不良反应而应用巴利昔单抗预防急性GVHD的17例难治性恶性血液病患者。男7例，女10例，中位年龄43（18～67）岁。停用CNI后，给予巴利昔单抗1 mg/kg每周1次进行替代预防，直至患者有条件重新启用CNI或哺乳动物雷帕霉素靶体蛋白（mTOR）抑制剂。

**结果:**

巴利昔单抗的替代预防中位开始时间为移植后5（1～32）d，替代治疗中位持续时间为20（7～120）d。所有患者均获得粒细胞植入，中位植入时间12（10～17）d。13例患者获得血小板植入，中位植入时间13（11～20）d。8例（47.1％）患者发生Ⅱ～Ⅳ度急性GVHD，4例（23.6％）发生Ⅲ/Ⅳ度急性GVHD。仅有1例患者死于急性GVHD。至随访截止，17例患者中7例死亡，存活患者中随访最长时间为347 d，中位生存时间未达到，移植后6个月总生存率为62.6％。17例患者中，13例（76.4％）发生巨细胞病毒再激活，7例（41.2％）发生EB病毒激活，未发生巨细胞病毒病。

**结论:**

在allo-HSCT过程中出现CNI不耐受时，短期单药应用巴利昔单抗可作为急性GVHD的替代预防方案。

## Abstract (en)

**Objective:**

To investigate the efficacy of short-term substitution of recombinant humanized anti-CD25 monoclonal antibody (Basiliximab) as acute GVHD (aGVHD) prophylaxis in calcineurin inhibitors (CNI) intolerant patients following allogeneic hematopoietic stem cell transplantation (allo-HSCT).

**Methods:**

This study included 17 patients with refractory malignant hematological disorders who underwent salvage allo-HSCT at the Bone Marrow Transplantation Department of Shanghai Zhaxin Traditional Chinese and Western Medicine Hospital from August 2021 to August 2022 and were treated with Baliximab to prevent aGVHD due to severe adverse reactions to CNI. There were seven men and ten women, with a median age of 43 years (18−67). Following the discontinuation of CNI, Basiliximab was administered at a dose of 1 mg/kg once weekly until CNI or mTOR inhibitors were resumed.

**Results:**

Basiliximab was started at an average of 5 (1−32) days after HSCT. The median duration of substitution was 20 (7−120) days. All had neutrophil engraftment within a median of 12 (10−17) days. Thirteen patients had platelet engraftment after a median of 13 (11−20) days. Four patients did not develop stable platelet engraftment. Eight patients (47.1％) developed Grade Ⅱ-Ⅳ aGVHD, while four (23.6％) developed Grade Ⅲ/Ⅳ aGVHD. Only one patient died from aGVHD. Before the end of the followup period, seven of 17 patients died. The longest followup period of the survivors was 347 days, and the median survival rate was not met. The overall survival (OS) rate at six months was 62.6％. Among the 17 patients, 13 (76.4％) experienced cytomegalovirus reactivation, 7 (41.2％) experienced EB virus activation, and no cytomegalovirus disease was observed.

**Conclusion:**

When CNI intolerance occurs during allo-HSCT, short-term replacement with Baliximab can be used as an alternative to prevent aGVHD.

异基因造血干细胞移植（allo-HSCT）是治疗难治性恶性血液病患者的重要手段[1]。移植物抗宿主病（GVHD）是移植后严重并发症之一。到目前为止，钙调蛋白抑制剂（CNI）仍然是最重要的急性GVHD预防药物。但是临床上有许多患者因药物不良反应不能耐受CNI，包括急性肝肾损伤、中枢神经系统并发症（CNSC）、血管内皮损伤综合征等[2]。停药是应对CNI不良反应的首选方案[3]–[5]，然而移植早期停用CNI有诱发严重急性GVHD的风险。因此，探索安全有效的CNI替代方案尤为重要。重组抗CD25人源化单克隆抗体巴利昔单抗（Basiliximab）通过竞争性结合活化T细胞上的IL2受体起到抑制活化T细胞作用，目前主要用于糖皮质激素耐药急性GVHD的治疗[6]–[9]。在实体器官移植中，已有重组抗CD25人源化单克隆抗体单药成功替代CNI的相关报道。在造血干细胞移植中，出现严重CNI相关不良反应能否同样应用重组抗CD25人源化单克隆抗体进行替代呢？为此，我们启动了一项探索性临床研究，对出现严重CNI相关不良反应的allo-HSCT患者短期应用巴利昔单抗单药进行GVHD预防，观察其有效性及安全性。

## 病例与方法

一、病例资料

2021年8月至2022年8月间，上海闸新中西医结合医院骨髓移植科行挽救性allo-HSCT治疗的难治性恶性血液病患者中，因严重不良反应不能耐受CNI而进行巴利昔单抗替代预防的患者17例。均随访至2022年12月31日。其中男7例，女10 例，中位年龄43（18～67）岁。入选患者中，急性髓系白血病（AML）5例，外周T细胞淋巴瘤（PTCL）5例，骨髓增生异常综合征（MDS）3例，慢性粒-单核细胞白血病（CMML）2例，原发性骨髓纤维化（PMF）白血病转化1例，T淋巴母细胞淋巴瘤1例。17例患者均为挽救性移植，移植前一般情况较差。17例患者中，11例造血干细胞移植合并症指数（HCT-CI）1分，5例HCT-CI为2分，1例PMF患者因胆红素升高及感染评为4分。11例（64.7％）患者ECOG评分≥2分，6例（47.5％）ECOG评分≥3分，卧床时间超过50％。2例ECOG评分为4分。

二、供者情况

无关供者造血干细胞移植（UD-HSCT）1例，单倍体造血干细胞移植（haplo-HSCT）16例。8例为男性供者（子供父母5例，父供子女者3例），9例为女性供者（女儿供父母6例，母供子1例，姐供弟1例）。供者中位年龄32（14～68）岁。供患者血型一致8例，血型不同9例。

三、预处理方案

所有患者均接受清髓性预处理。AML、MDS、CMML患者以下预处理方案：白消安3.2 mg·kg^−1^·d^−1^，连用2～4 d；克拉屈滨5 mg·kg^−1^·d^−1^或氟达拉滨30 mg·kg^−1^·d^−1^，连用5 d；阿糖胞苷1～2 g·m^−2^·d^−1^，连用5 d；部分患者加用全身放射治疗（TBI）3 Gy。淋系肿瘤（PTCL、T淋巴母细胞淋巴瘤）患者予以TBI为基础的预处理方案（TBI 10 Gy，依托泊苷30 mg/kg，环磷酰胺 100～120 mg/kg）。

四、干细胞回输

采用粒细胞集落刺激因子（G-CSF）进行供者干细胞动员。采集完成后立即回输。中位有核细胞回输量为17.89（7.38～33.51）×10^8^/kg，中位CD34^+^细胞回输量10.58（6.09～19.29）×10^6^/kg，中位CD3^+^细胞回输量为3.04（1.09～4.66）×10^8^/kg。

五、GVHD的预防及治疗

急性GVHD诊断参照NIH诊断分级标准[10]。

所有患者的初始GVHD预防方案均为CNI联合短程甲氨蝶呤（MTX）及抗胸腺细胞球蛋白（ATG）方案：环孢素A 2 mg/kg或他克莫司0.02 mg/kg持续静脉泵入（移植前2 d起），维持环孢素A血药浓度200～300 µg/L或他克莫司血药浓度5～10 µg/L；MTX：移植后第1天15 mg/m^2^，移植后第3、6天各予以10 mg/m^2^；T细胞淋巴瘤患者采用兔抗人胸腺细胞球蛋白（rATG）2.5 mg·kg^−1^·d^−1^×4 d，其余患者均采用rATG 2 mg·kg^−1^·d^−1^×3 d。

发生急性GVHD后，常规加用甲泼尼龙0.5～1 mg·kg^−1^·d^−1^并联合芦可替尼5 mg每日2次进行治疗。

六、CNI不良反应及处理

CNI不良反应包括肾小球滤过率下降超过50％并有持续下降趋势、总胆红素超过正常值上限两倍以上或有明显上升趋势、发生CNSC（包括癫痫发作、意识丧失、不能控制的高血压脑病）、临床诊断为血管内皮损伤综合征。患者出现CNI不良反应后立即停用CNI，给予巴利昔单抗1 mg/kg每周1次进行替代预防，直至有条件重新启用CNI或西罗莫司。

七、病毒监测及治疗

应用实时定量聚合酶链反应监测外周血巨细胞病毒（CMV）、EB病毒（EBV）以及人类疱疹病毒6型（HHV-6）载量。检测阈值为1 000拷贝/ml。移植后1个月内常规每周3次进行外周血病毒载量监测。移植后2个月至100天每周至少进行1次外周血病毒检测。出现腹泻时常规进行粪便病毒监测。出现脑病的患者进行HHV-6监测。外周血CMV-DNA阳性患者，予更昔洛韦或者膦甲酸治疗直至病毒DNA阴性，HHV-6脑炎患者予以膦甲酸治疗。

八、随访

采用查阅住院/门诊病历以及电话随访方式进行随访。随访截至2022年12月31日。移植后总生存（OS）时间定义为造血干细胞回输完毕至随访终点或死亡的时间。

九、统计学处理

数据采用SPSS26.0软件进行处理。采用Kaplan-Meier方法计算OS率。

## 结果

一、植入情况

所有患者均获得粒细胞植入，中位植入时间12（10～17）d。13例患者血小板植入，中位植入时间为13（11～20）d。4例患者血小板未植入，其中3例为早期死亡患者，1例为移植相关血栓性微血管病（TA-TMA）后植入不良患者。除了1例供者特异性抗体（DSA）强阳性患者在移植后1个月内死亡而未获完全植入外，其余患者均在移植后14 d内达到完全供者嵌合。

二、CNI不良反应及替代预防

本研究纳入了17例出现严重CNI不良反应的患者。5例（29.4％）患者肾、肝等单个脏器功能严重不全（肾脏4例，肝脏1例），5例（29.4％）为CNSC（癫痫发作、意识障碍、不能控制的高血压脑病），4例（23.5％）为两个以上脏器功能不全（3例为肝肾功能不全，1例为肾功能不全合并CNSC），3例（17.64％）为内皮损伤综合征［1例TA-TMA，1例肝窦阻塞综合征（SOS），1例渗漏综合征］。

巴利昔单抗的替代预防剂量为1 mg/kg每周1次，替代预防中位开始时间移植后5（1～32）d，替代治疗中位持续时间20（7～120）d，替代治疗中位次数为3（1～13）次。除1例患者外，余16例患者的替代预防次数均不超过4次。14例患者在度过危重时期，状态稳定后，重新加用CNI或者西罗莫司进行免疫抑制治疗。3例患者后续未加用CNI或西罗莫司。其中，1例患者因反复肝功能不全持续应用巴利昔单抗替代；1例为早期死亡患者；1例为TA-TMA患者，替代预防3次后无急性GVHD发生，未再加用免疫抑制剂。

三、急性GVHD发生情况

在17例替代预防患者中，8例（47.1％）发生Ⅱ～Ⅳ度急性GVHD，4例（23.6％）发生Ⅲ/Ⅳ度急性GVHD，仅有1例患者死于急性GVHD（ECOG评分4分，预处理中因肝肾功能不全予巴利昔单抗替代预防，因肝脏、肠道Ⅳ度急性GVHD不能控制死亡）。2例患者替代预防后发生植入综合征。其中1例患者后续发生Ⅲ度急性GVHD，经抗急性GVHD治疗后好转。

四、生存及复发

随访截至2022年12月31日，17例患者的中位随访时间为128 d，存活患者中最长随访时间为347 d，中位OS时间未达到，移植后6个月OS率为62.6％（图1）。17例患者中，7例死亡，其中3例在移植后100 d内死亡，仅有1例死于急性GVHD，急性GVHD的死亡率为5.8％。另外2例分别死于SOS和多脏器功能不全。移植100 d后死亡4例，1例因反复肺感染继发心、肾功能不全死亡，1例因TA-TMA死亡，余2例患者死于原发病复发。

五、病毒感染情况

17例患者中有13例（76.4％）发生CMV再激活，均予更昔洛韦或膦甲酸抗病毒后好转，未发生CMV病。7例（41.2％）患者发生EBV再激活，除1例患者考虑发生EBV感染相关TA-TMA予利妥昔单抗治疗外，其余患者予观察随访后转阴。2例（11.76％）患者发生HHV-6感染（1例为HHV-6脑炎，予以膦甲酸治疗后好转；另1例为HHV-6病毒性肠炎，予以更昔洛韦治疗后好转）。

**图1:**
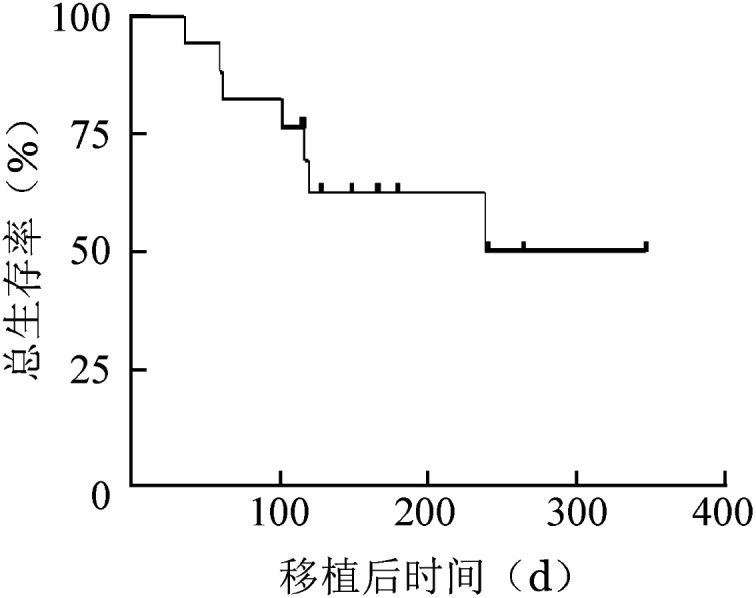
17例应用巴利昔单抗预防移植物抗宿主病恶性血液病患者挽救性异基因造血干细胞移植后总生存曲线

## 讨论

接受挽救性移植的恶性血液病患者常经过多线化疗，移植后脏器功能不全及严重并发症发生率明显增高。一项纳入84例复发难治AML患者（中位年龄61岁）的研究结果显示，HCT-CI和ECOG评分对移植后OS均有明显影响。其中ECOG评分是独立预后因素，≥2分的患者移植后1年OS率不足30％，脏器功能不全是这类患者的主要死亡原因之一[11]。如何减少移植早期并发症，降低移植相关死亡是这类患者的治疗难点。

CNI是预防GVHD的重要药物，在部分患者中可发生急性肝肾功能不全、CNSC以及血管内皮损伤综合征等严重不良反应[12]。这些CNI的并发症均导致移植风险增加，患者死亡率明显上升。Tokgoz等[3]研究表明，移植后急性肾损伤的高危时机为移植后2～3周，CNI水平与重度急性肾损伤密切相关。而CNI本身又可能损害血管内皮而导致SOS、TA-TMA等加重肾功能损伤[12]–[13]。肾功能损害患者3个月内死亡率超过30％[13]–[15]。TA-TMA等血管内皮损伤综合征作为移植后非常严重的并发症，报道的死亡率接近100％[16]–[18]。移植早期非感染相关的CNSC发生率报道高低不一，但发生CNSC患者的生存期均明显缩短[19]–[21]。在一项纳入191例allo-HSCT患者的研究中，CNSC的发生率为14％，40％的患者在移植后30 d内死亡，移植后1年非复发死亡率达42％[21]。在另一项纳入153例allo-HSCT患者的研究中，CNSC的发生率为22％，80％以上的CNSC发生于移植后100 d内，其中约50％与CNI有关[20]。CNI均是以上各种并发症的重要诱因之一，积极撤停CNI有助于并发症的治疗。然而该时期恰好也是急性GVHD发生的高危期，如何在停用CNI的同时避免严重急性GVHD发生是迫切需要解决的问题。目前国内外尚无标准CNI替代治疗方案。巴利昔单抗无血管内皮损伤，也无肾毒性及神经毒性，是潜在的优选替代药物[22]。在实体器官移植中，已有成功应用CD25单抗替代CNI的小规模临床报道。Cantarovich等[23]在肾移植的研究中，应用巴利昔单抗替代CNI，在保证植入的同时保护肾脏功能，减少患者的透析需求。Kirchner等[24]在CNI不耐受的胰腺移植患者中应用达利珠单抗作为移植后免疫抑制剂，相较于对照组，移植物和患者的存活率均更高。在造血干细胞移植中，仅有Wolff等[22]报道了一项纳入18例患者的临床研究。该研究在伴有GVHD的TA-TMA及肾功能不全患者中应用达利珠单抗替代CNI进行急性GVHD治疗，部分患者在获得肾功能改善的同时急性GVHD也得到有效控制。巴利昔单抗单药替代CNI进行急性GVHD预防尚未见报道。故而我们中心启动了移植早期应用巴利昔单抗单药替代CNI进行GVHD预防的探索性临床研究，观察了该组患者替代预防的疗效及治疗结局。本组病例替代预防开始中位时间为移植后5（1～32）d，替代预防中位持续时间为20（7～120）d。替代治疗时间段覆盖了移植早期脏器功能不全、中枢神经系统异常、内皮损伤综合征等移植相关并发症的高发时间窗。本研究因脏器功能不全进行替代预防的9例患者中，6例（66.7％）替代预防后脏器功能恢复；发生血管内皮损伤综合征的3例患者中，2例经替代预防后好转，均在恢复后顺利重新启用CNI。所有患者移植后6个月OS率为62.6％。因CNSC而停用CNI予巴利昔单抗替代预防的5例患者中，1例继发TA-TMA死亡外，其余4例（80％）均在症状缓解后恢复CNI应用，且后续未再出现CNSC症状，在随访期内均存活，生存情况优于文献[21], [25]报告的结果。因此，我们的研究为CNI撤停期间提供了安全、有效的急性GVHD替代预防方案。

尽管移植技术不断进步，新药不断出现，急性GVHD仍然是移植后重要并发症。目前不同研究中，急性GVHD发生率为30％～50％[26]。CNI作为急性GVHD预防的经典药物仍然不可或缺。不含CNI的GVHD预防方案仅有小样本临床报道且结果均不尽如人意。Shkalim-Zemer的研究中，在14例发生CNSC的allo-HSCT患者中尝试停用CNI并改用雷帕霉素、霉酚酸酯、糖皮质激素多药联合方案进行急性GVHD预防，6例（42％）死于严重急性GVHD，1例存活患者发生Ⅳ度 急性GVHD[25]。本研究中，4例（23.6％）患者发生Ⅲ/Ⅳ度急性GVHD，仅有1例因急性GVHD死亡。在含CNI、霉酚酸酯及MTX的经典GVHD预防方案的haplo-HSCT中，黄晓军教授团队报道Ⅱ～Ⅳ度急性GVHD发生率为40％～46％，Ⅲ/Ⅳ度急性GVHD发生率为11％～17％[27]。本组病例Ⅱ～Ⅳ度急性GVHD发生率为47.1％，与之基本持平，Ⅲ/Ⅳ度急性GVHD发生率为23.6％，较上述研究稍高。本研究纳入的均为进展期挽救治疗患者，除T细胞淋巴瘤患者外，均应用ATG 6 mg/kg预防治疗，较低的ATG剂量可能也是急性GVHD发生率稍偏高的原因[28]。本研究中，在随访期内仅有1例患者因GVHD死亡，表明在CNI停用期间仅予巴利昔单抗单药替代预防急性GVHD是安全的。

本组病例CMV血症的发生率为76.5％（13/17），与各研究中报道的haplo-HSCT后CMV血症发生率73％～83％类似[29]–[31]；EBV血症发生率为41.2％（7/17），与报道的allo-HSCT后EBV血症发生率27％～45％也基本一致[31]–[32]。

有研究表明，CD25单抗可下调调节性T细胞表达，从而导致停药后GVHD复发或慢性GVHD发生[33]–[34]。本组病例100 d以后存活的患者中，除1例持续应用巴利昔单抗替代治疗外，其余患者均在并发症控制后转为以CNI为基础的经典GVHD控制方案。因此，巴利昔单抗短期替代治疗对慢性GVHD发生的影响尚需继续研究。
